# Surgical management of multilevel cervical spinal stenosis and spinal cord injury complicated by cervical spine fracture

**DOI:** 10.1186/s13018-014-0077-4

**Published:** 2014-08-22

**Authors:** Zhao-Wan Xu, Deng-Xing Lun

**Affiliations:** 1 Department of Spine Surgery, Weifang People’s Hospital, Guangwen Road, Kuiwen District, Weifang 261041, Shandong, China

**Keywords:** Laminoplasty, Spinal cord injury, Spinal stenosis

## Abstract

**Background:**

There are few reports regarding surgical management of multilevel cervical spinal stenosis with spinal cord injury. Our purpose is to evaluate the safety and feasibility of open-door expansive laminoplasty in combination with transpedicular screw fixation for the treatment of multilevel cervical spinal stenosis and spinal cord injury in the trauma population.

**Methods:**

This was a retrospective study of 21 patients who had multilevel cervical spinal stenosis and spinal cord injury with unstable fracture. An open-door expansive posterior laminoplasty combined with transpedicular screw fixation was performed under persistent intraoperative skull traction. Outcome measures included postoperative improvement in Japanese Orthopedic Association (JOA) score and incidence of complications.

**Results:**

The average operation time was 190 min, with an average blood loss of 437 ml. A total of 120 transpedicular screws were implanted into the cervical vertebrae between vertebral C3 and C7, including 20 into C3, 34 into C4, 36 into C5, 20 into C6, and 10 into C7. The mean preoperative JOA score was 3.67 ± 0.53. The patients were followed for an average of 17.5 months, and the average JOA score improved to 8.17 ± 1.59, significantly higher than the preoperative score (*t* = 1.798, *P* < 0.05), with an average improvement of 44.7 ± 11.7%. Postoperative complications in four patients included cerebrospinal fluid leakage, delayed wound healing, pulmonary infection, and urinary system infection. All four patients were responsive to antibiotic treatment; one died from respiratory failure 3 months postoperatively.

**Conclusions:**

The open-door expansive laminoplasty combined with posterior transpedicular screw fixation is feasible for treating multilevel cervical spinal stenosis and spinal cord injury complicated by unstable fracture. Its advantages include minimum surgical trauma, less intraoperative blood loss, and satisfactory stable supportive effect for reduction of fracture.

## Introduction

Operative treatment of cervical spinal stenosis remains controversial [1]. Several options are commonly used, including anterior subtotal corpectomy combined with bone graft fusion and internal fixation [2],[3], anterior discectomy combined with bone graft fusion and internal fixation [4],[5], and posterior laminoplasty with or without internal fixation [6]. However, there are limitations with these options [1],[6]. For instance, the multiple cervical vertebrae fusion with a large bone graft through anterior route could lead to severe disability of cervical mobility or poor fusion and severe complications, such as dysphagia and dyspnea [7]-[9]. On the other hand, posterior laminoplasty is a relatively simple operation, which could preserve cervical mobility with fewer postoperative complications; therefore, posterior laminoplasty has become one of the most effective approaches for multilevel cervical spinal stenosis.

However, there is a lack of clinical study on the surgical strategy for multilevel cervical spinal stenosis and spinal cord injury complicated by unstable fracture. Although complete decompression could be achieved for spinal stenosis by posterior laminoplasty, the deterioration on the cervical vertebrae due to surgery could aggravate the instability of cervical vertebrae [2],[6]. In contrast, the stability of cervical vertebrae could be reconstructed by anterior surgery, but at the cost of insufficient spinal decompression and more postoperative complications [3]. Therefore, to fulfill the requirements of both complete decompression and satisfactory stability of reconstruction is one of the challenges in clinical practice. The purpose of the current study was to evaluate the safety and feasibility of open-door expansive laminoplasty in combination with transpedicular screw fixation via posterior route for the treatment of multilevel cervical spinal stenosis complicated by cervical spine fracture.

## Materials and methods

### Patients

We enrolled 170 patients with cervical vertebrae trauma who presented to our hospital between January 2010 and March 2012 for retrospective analysis. There were 21 patients with multilevel cervical spinal stenosis and spinal cord injury complicated by unstable fracture, which were diagnosed according to CT and MRI findings. Their ages ranged between 51 and 69 years (average 55.7 years), and the male-to-female ratio was 15:6. Twenty-one patients were further classified according to disease type: two patients had posterior longitudinal ligament ossification concurrent with one intervertebral disc rupture; four patients had two disc herniation and one rupture; four patients had two herniation and two ruptures; and 11 patients had three herniation and three ruptures. SLIC (subaxial cervical spine injury classification) points of all patients were more than 5 points (mean 5.9 points).

### Inclusion criteria

Inclusion criteria were as follows: (1) cervical spinal stenosis across at least two cervical segments; (2) unstable cervical fracture without dislocation, such as the concurrent intervertebral disc ruptures or posterior longitudinal ligament injury; and (3) kyphotic deformity could be included.

### Exclusion criteria

Exclusion criteria were as follows: (1) single-segment cervical stenosis, (2) cervical fracture with dislocation or traumatic cervical disc herniation, and (3) severe disc herniation which cannot be alleviated by the surgery via posterior route.

### Surgical approach

The patient was placed in a prone position with the head positioned in a U-shape support, and surgery was performed under general anesthesia with tracheal intubation. Skull traction, with the force of 5 kg, was sustained throughout the surgery. The skin at the surgical site was pulled caudally from the shoulders by a pair of wide medical adhesive plasters, eliminating the skin wrinkles to facilitate the operation. The posterior median incision was made using electric knife to dissect layer-by-layer from the skin to the spinous process along the median line of the ligament. The bilateral paravertebral muscles of the articular process were dissociated to expose the involved spinous process, lamina, and facet joints. The bone of the screw-targeted site was drilled according to the anatomic landmarks and preoperative transpedicular computed tomographic images, and a handmade aiming apparatus was placed on the spinous process just above or below the screw-targeted segment. Drills of 2-mm diameters were inserted through the cannula of the aiming device, and the cannula was adjusted according to the designed screw-implementation process to move the drill steadily through the pedicle to the vertebral body. The screw with appropriate diameter was implanted into the callous bone under the guidance of a detector within the cannula. The connecting rod must be pre-bended according to the physiological curvature of cervical vertebra before implementation, where distraction and reduction might be needed for screw locking. Afterward, open-door expansive spinal decompression via posterior route was performed along the cervical vertebrae C3 to C7, and the suspended laminae were fixed to the connecting rod.

Skull traction was removed after surgery, and the routine postoperative medical treatments were carried out, including glucocorticoid administration, dehydration treatment, conventional nerve nutrition, and prophylactic antibiotic treatment. In addition, functional rehabilitation exercise at an early stage of recovery was advised to all patients.

### Evaluation criteria

The Japanese Orthopedic Association (JOA) scoring system was used to evaluate functional recovery before and immediately after surgery and at the final follow-up. The recovery rate was calculated according to the following equation: recovery rate (%) = (postoperative score–preoperative score) × 100/(full score–preoperative score). Surgical time, blood loss volume, and the occurrence of surgical complications were also investigated.

**Figure 1:**
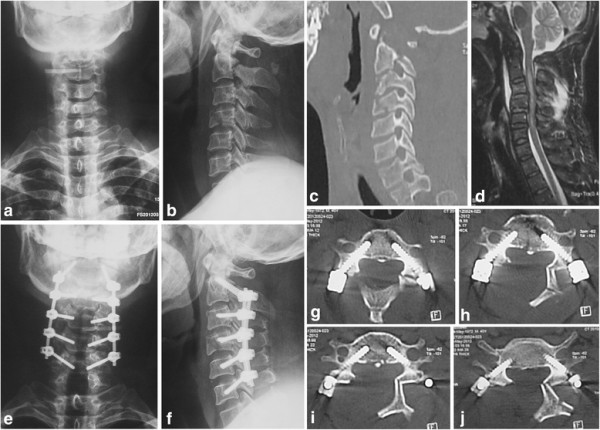
**Radiographic studies of the cervical defects and performed procedures. (a–c)** Preoperative anteroposterior images of vertebral fracture in cervical vertebrae C2 and C3. **(d)** Spinal stenosis between C3 and C4, and between C4 and C5 are shown with preoperative magnetic resonance imaging; spinal cord deformation, posterior ligament complex injury, and hematoma anterior to vertebrae are revealed between C2 and C5. **(e–f)** Open-door posterior laminoplasty and transpedicle internal fixation at C2–C5. **(g–j)** Transpedicle screws were embedded into the pedicle.

The safety of CPS was evaluated by radiological results (Figure 1). The implemented screw was classified into four types according to the position of the screw [10]. Type I was classified as the screw being completely inserted into the pedicle or pedicle cortex or no perforation; type II, perforation of less than one fourth of the screw diameter; type III, perforation of between one fourth and one half of the screw diameter; type IV, perforation of 50% of the screw diameter or more. In the current study, types I and II were regarded as an ‘excellent status’.

### Statistical analysis

The data were presented as mean ± standard deviation (SD) and analyzed using software SPSS12.0. The independent-sample *t* test was utilized for comparison between pedicle axis tilt angle and screw angle. *P* < 0.05 was considered as the statistical significance.

## Results

### General information

**Figure 2:**
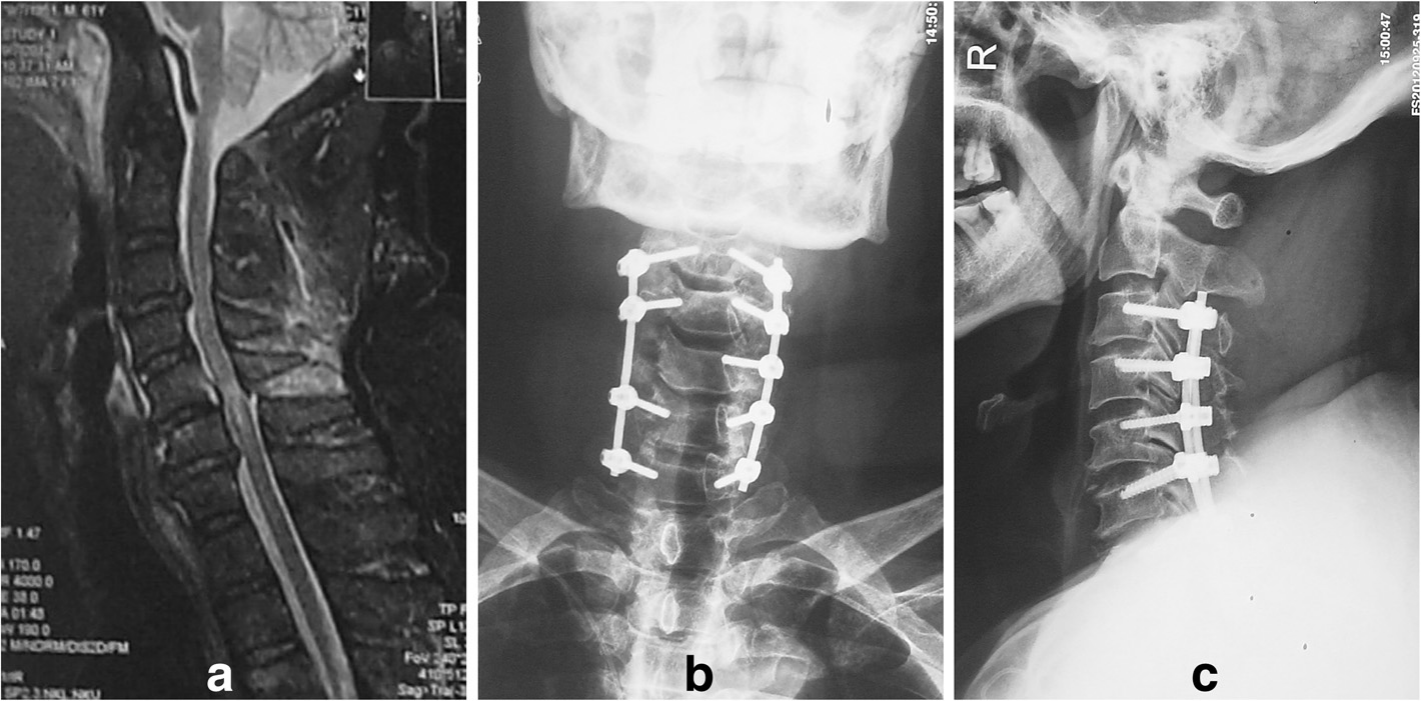
**Radiologic studies of spinal stenosis and open-door posterior laminoplasty and transpedicle internal fixation. (a)** Spinal stenosis between C3 and C4, C5 and C6, and C6 and C7 are shown with preoperative magnetic resonance imaging, in combination with posterior ligament complex injury. **(b, c)** Open-door posterior laminoplasty and transpedicle internal fixation at C3–C7.

Internal fixation was performed on all 21 patients: seven had fixation at two vertebrae (four screws), ten had fixation across three vertebrae (six screws), and four had fixation across four vertebrae (eight screws), and one pedicle screw was missing because of the anatomic variation (Figure 2). Thus, 121 transpedicular screws were implanted: 20 screws into C3, 34 into C4, 36 into C5, 20 into C6, and ten into C7. The operation time was 120–250 min (average 190 min), and the average intraoperative blood loss was 320–870 ml (average 437 ml).

### Postoperative functional examination

All patients were followed up postoperatively: average follow-up period of 17.5 months (3–36 months). The preoperative JOA score was 3.67 ± 0.53 and improved to 8.17 ± 1.59 at the last follow-up examination, which was significantly higher than the mean preoperative score (*t* = 1.798, *P* < 0.05), with an average improvement of 44.7% ± 11.7%. The average JOA score of the seven paraplegic patients was 2.17 ± 0.33 before operation and improved to 3.16 ± 0.42 after operation (*t* = 1.798, *P* < 0.05). The preoperative JOA score was 4.42 ± 0.62 for the other 14 patients and improved to 10.68 ± 0.37 after operation (*t* = 10.12, *P* < 0.05).

### Complications

Postoperative complications were found in four patients, including one having cerebrospinal fluid leakage with delayed wound healing; three patients had pulmonary infection, including one with concurrent urinary infection. All patients were responsive to antibiotic treatment, but one died from respiratory failure 3 months after the operation.

According to the position of screw, 109 screws were of type I, ten were type II, one was type III, and none was type IV. In total, the excellence rating of the screw position was 99.2%. However, 11 patients had breakthrough screws, four with the inferior wall of vertebrae broken, three with the lateral wall broken, and four with a medial wall broken. No vascular or nerve injuries were found caused by the breakthrough of a screw. Neither loosening, dislocating, or fracture of internal fixation nor complication of nerve paralysis was observed.

## Discussion

Severe results are often observed in the patient with multilevel cervical stenosis complicated by cervical spine fracture. The canal space is reduced by stenosis, which may have already induced mild decompression symptoms, and the slightest violence could lead to edema or degeneration of nerve root or spinal cord, manifested as the deterioration in symptoms. Paralysis, disappearance of sense perception, or gatism are found among severely injured patients who usually have poor preoperative JOA score and no surgical therapeutic effect. Moreover, the prognosis of patients might be significantly affected by different surgical approaches. To achieve complete decompression of the spinal cord, restore nerve function as much as possible, and remain the local stability of cervical vertebra, and to reduce postoperative complications are the main aims of medical treatment for patients with multilevel cervical stenosis complicated by unstable cervical fracture [6].

### Decompression and complications

There are various options for surgical management of spinal cord decompression; however, neither conveys satisfactory outcomes with respect to postoperative complications and clinical therapeutic effect. For instance, a high rate of nonunion (17%–45%) has been found with the treatment of anterior cervical discectomy and fusion (ACDF) for multilevel cervical spondylotic myelopathy due to large bone graft [7],[8]. Surgery for bone graft fusion through both anterior and posterior routes is another option, but brings severe surgical trauma to the patients [11], and yet the stability may not be significantly improved [6]. The cervical posterior longitudinal ligament must be removed in laminectomy via the posterior route, which could interfere with the anatomical structure and lead to poor stability of cervical vertebrae. Therefore, posterior laminoplasty, rather than laminectomy, is preferential clinically.

On the other hand, although a smaller bone graft with high fusion rate and clear intraoperative operation field could be achieved by subtotal corpectomy and laminoplasty via an anterior route, poor postoperative stability and more complications are of concern, especially for multilevel cervical myelopathy. Complete decompression is advantageous with open-door posterior laminoplasty, due to better postoperative stability, as compared to subtotal corpectomy and laminectomy on two or more segments of cervical myelopathy. This is because less bone graft is used with an improved fusion rate; however, axial pain often results [1],[7],[12]. Meanwhile, although posterior transpedicular screw fixation conveys better stability [13], the narrowed operating room, complicated local anatomical structure, and large camber angle make the surgical manipulation difficult; a minor mistake during the operation would result in severe complications [13].

We concluded that anterior subtotal corpectomy and posterior open-door laminoplasty are more suitable for multilevel cervical spinal stenosis. The posterior operation offers better decompression and fewer complications and is preferred by patients. The posterior open-door technology was considered simple and offers better decompression, preserving cervical spinal mobility, in an analysis reported by Anthony et al. [14]. In addition, this approach has fewer postoperative complications and lower cost. For patients with complete paralysis, the anterior or anterior in combination with posterior approaches had higher rates of postoperative infection due to severe surgical trauma [11]. Besides, when Charles et al. [7] compared the therapeutic outcomes of subtotal corpectomy (*n* = 49) and laminoplasty (*n* = 40), they reported better functional improvement with laminoplasty, with less intraoperative blood loss (360 ml vs. 572 ml with subtotal corpectomy), fewer complications (1/40 vs. 9/49 with subtotal corpectomy), and a lower degeneration rate (8% vs. 38%). On the other hand, Shibuya et al. [12] compared therapeutic outcomes of anterior subtotal corpectomy (*n* = 49) and posterior laminoplasty (*n* = 40) and found that for multilevel vertebral lesions, the operation time was longer and intraoperative blood loss was greater by subtotal corpectomy, and complications such as disappearance of cervical physiological curvature and kyphosis were often found. Similarly, Wada et al. [1] found in a comparative study of corpectomy (*n* = 45) and posterior open-door laminoplasty (*n* = 41) that although the cervical functional improvement (JOA score) was not significantly different between the two surgical approaches, a higher rate of degeneration in adjacent vertebra was found with posterior laminoplasty with deteriorated symptoms [2],[3]. In addition, shorter operation time and less intraoperative blood loss were found with laminoplasty (182 min and 608 g by laminoplasty vs. 264 min and 986 g by subtotal corpectomy). As for postoperative complications, Kazuo et al. [15] found that the complication rate was 29.3% by anterior subtotal corpectomy and 7.1% by posterior open-door laminoplasty for the patients with multilevel cervical spinal stenosis. Based on these published reports, we suggest posterior open-door laminoplasty as the primary approach for multilevel cervical spinal stenosis, in agreement with Yang et al. [16]. In our study, we found that the operation time was 143.6 ± 31.7 min vs. 116.5 ± 29.8 min, intraoperative blood loss was 107.5 ± 49.6 ml vs. 172.3 ± 68.2 ml, and postoperative complication rates were 21.7% vs. 43.6% for ACDF and ACCF, respectively. Therefore, we propose open-door laminoplasty is more suitable for patients with multilevel cervical spinal stenosis.

However, there is a limitation with single open-door laminoplasty, such as high rates of axial pain [1],[7],[12], due to disuse atrophy and ischemia of neck muscles, and delayed healing process of the articular processes. For instance, it was found that the rate of postoperative axial pain was higher in open-door laminoplasty by Wada et al. [1], along with limited cervical motility. Therefore, patients were usually instructed to use cervical support to avoid the axial pain and to do rehabilitation exercises at an early stage to prevent local muscle ischemia [7]. Similarly, Wada et al. suggested axial lateral bone graft to reduce bone nonunion rate and neck-supportive protection for 3 weeks to prevent muscle atrophy, ischemia, or bone nonunion. In the current study, there was no axial pain found, which might be due to the strong support of internal fixation by transpedicle screws, facilitating early rehabilitation exercise and therefore effectively reduced the axial pain.

### Biomechanical properties

The stability reconstruction is one of the main purposes of spinal operation, especially for patients with unstable spine fracture. The posterior internal fixation approach could offer better postoperative spinal stability than the anterior approach [17]. White et al. [18] suggested, from the biomechanics perspective, that the anterior internal fixation should be used for one or two segments of cervical spinal stenosis, the posterior approach should be used for three or more segments, and posterior decompression in combination with articular process fusion should be used for patients with unstable cervical vertebrae. Moreover, DiAngelo et al. [19] suggested that subtotal corpectomy and graft bone fusion might not provide sufficient stability for multilevel myelopathy. In addition, Do Koh et al. [10] utilized ten models of cadaveric bone for the study of stability reconstruction for cervical spine fracture with dislocation and vertebral burst fractures, and it was found that better stability could be achieved by a posterior lateral screw fixation technique, as compared with anterior steel plate fixation. Moreover, they suggested that there should be strong external support when single anterior internal fixation was used, especial for patients with longitudinal ligament injuries.

The posterior transpedicle screw internal fixation has been shown to convey better stability for unstable spinal fracture, including spinal fracture with dislocation, than anterior discectomy in combination with one graft fusion [11],[20]. In addition, it was confirmed by Nakashima et al. [11] that satisfactory therapeutic outcomes and stable bone fusion were achieved for 40 patients with cervical fracture with dislocation and traumatic disc herniation, by single posterior transpedicular screw fixation. We also showed that posterior internal fixation was superior to anterior decompression in terms of postoperative stability, and it avoided the risk of exacerbation of neural symptoms [11]. The transpedicle screw was outstanding among the posterior internal fixation approaches, because of its three-column stabilization property, with superior biomechanical properties to lateral mass screws and spinous process wire fixation. For instance, it was demonstrated by Kotani et al. [21] that the stability of transpedicle internal fixation was much superior to other internal fixations via either posterior or anterior routes and was especially suitable for patients with multilevel unstable spinal fractures. Moreover, Jones et al. [22] showed that pullout forces were greater for transpedicle screws than for lateral mass screws, implying better stability of posterior internal fixation; transpedicle screw internal fixation was also more suitable for patients with multilevel spinal stenosis with unstable fractures.

## Conclusion

Open-door posterior laminoplasty is the most efficient approach from the perspective of complete spinal decompression, and posterior transpedicle screw internal fixation is the best from the perspective of local postoperative stability. Therefore, the open-door expansive laminoplasty in combination with posterior transpedicular screw fixation was used in the current study, and our findings suggest that it is safe and feasible for patients with multilevel stenosis and unstable fracture.

## Competing interests

The authors declare that they have no competing interests.

## Authors' contributions

LDX and XZW participated in the design of this study, and they both collected important background information and drafted the manuscript. Both authors read and approved the final manuscript.
